# Deep cross entropy fusion for pulmonary nodule classification based on ultrasound Imagery

**DOI:** 10.3389/fonc.2025.1514779

**Published:** 2025-04-04

**Authors:** Xian Wang, Ziou Zhao, Donggang Pan, Hui Zhou, Jie Hou, Hui Sun, Xiangjun Shen, Sumet Mehta, Wei Wang

**Affiliations:** ^1^ Department of Ultrasound, Affiliated People’s Hospital of Jiangsu University, Zhenjiang, Jiangsu, China; ^2^ Medical College of Yangzhou University, Yangzhou, Jiangsu, China; ^3^ Department of Radiology, Affiliated People’s Hospital of Jiangsu University, Zhenjiang, Jiangsu, China; ^4^ Department of Pathology, Affiliated People’s Hospital of Jiangsu University, Zhenjiang, Jiangsu, China; ^5^ School of Computer Science & Communication Engineering, Jiangsu University, Zhenjiang, Jiangsu, China; ^6^ Department of Radiology, Affiliated Hospital of Yangzhou University, Yangzhou, Jiangsu, China

**Keywords:** pulmonary nodule classification, deep learning, ultrasound imagery, convolutional neural networks, cross-entropy fusion

## Abstract

**Introduction:**

Accurate differentiation of benign and malignant pulmonary nodules in ultrasound remains a clinical challenge due to insufficient diagnostic precision. We propose the Deep Cross-Entropy Fusion (DCEF) model to enhance classification accuracy.

**Methods:**

A retrospective dataset of 135 patients (27 benign, 68 malignant training; 11 benign, 29 malignant testing) was analyzed. Manually annotated ultrasound ROIs were preprocessed and input into DCEF, which integrates ResNet, DenseNet, VGG, and InceptionV3 via entropy-based fusion. Performance was evaluated using AUC, accuracy, sensitivity, specificity, precision, and F1-score.

**Results:**

DCEF achieved an AUC of 0.873 (training) and 0.792 (testing), outperforming traditional methods. Test metrics included 71.5% accuracy, 70.69% sensitivity, 70.58% specificity, 72.55% precision, and 71.13% F1-score, demonstrating robust diagnostic capability.

**Discussion:**

DCEF’s multi-architecture fusion enhances diagnostic reliability for ultrasound-based nodule assessment. While promising, validation in larger multi-center cohorts is needed to address single-center data limitations. Future work will explore next-generation architectures and multi-modal integration.

## 1 Introduction

Lung cancer, one of the most lethal forms of cancer worldwide, presenting a daunting challenge to public health systems across the globe (1). This disease’s insidious nature often allows it to progress undetected until it has reached an advanced stage, largely due to its tendency to develop without obvious or early symptoms. This silent progression makes early diagnosis crucial for improving patient outcomes, as detecting lung cancer in its early stages can vastly increase the chances of successful treatment.

Early lung cancer detection frequently involves identifying pulmonary nodules, small, rounded growths in the lungs. These nodules can be detected through imaging techniques and often serve as the first visible signs of potential lung cancer. However, it’s important to note that not all pulmonary nodules are cancerous; many are benign and pose no immediate health risk (2). Accurately distinguishing between benign and malignant nodules is crucial for timely clinical decisions and patient outcomes (3). Traditional imaging techniques often struggle to differentiate between these two types due to their overlapping characteristics.

Various imaging techniques have been employed for the detection and characterization of pulmonary nodules. In particular, computed tomography (CT) scans have revolutionized lung cancer screening programs due to their high sensitivity and specificity (4). CT scans can detect even small nodules that may be invisible on conventional chest X-rays. However, despite the advantages of CT scans in lung cancer screening, there are notable limitations. One significant drawback is the exposure to ionizing radiation, which carries potential health risks, especially for patients who require frequent monitoring or follow-up scans. Additionally, CT scans are expensive and may not be readily available in all healthcare settings, particularly in regions with limited medical infrastructure (5). These factors highlight the need for alternative imaging methods that can complement or, in some cases, replace CT scans in certain contexts.

Ultrasound imaging, on the other hand, offers several advantages over CT scans. It is non-invasive, does not involve ionizing radiation, and is relatively inexpensive. Ultrasound imaging provides real-time visualization of the lungs and surrounding structures, making it a valuable tool for image-guided interventions, such as percutaneous biopsies. Prior studies have demonstrated that ultrasound-guided biopsies are particularly effective for nodules larger than 3 cm, enhancing procedural accuracy and safety (6, 7). Research by Sabirovna and Raykhona (6) has shown that ultrasound is a reliable technique for guiding interventions in medium-to-large nodules, particularly in cases where CT imaging is not feasible due to accessibility or radiation concerns. Additionally, Brown et al. (7) emphasize that ultrasound-guided procedures are most beneficial for nodules exceeding 3 cm, as real-time imaging improves biopsy precision and reduces procedural risks. These findings reinforce the clinical utility of ultrasound in nodule evaluation and interventional guidance.

In recent years, advancements in artificial intelligence (AI) and deep learning have begun to revolutionize medical imaging (8). The integration of computer-aided detection (CAD) systems with imaging technologies like ultrasound has opened new avenues for improving the accuracy of nodule classification (9). Deep learning techniques, such as convolutional neural networks (CNNs), have shown great promise in processing complex image data and identifying subtle patterns that may be missed by human eyes. These technologies enable the extraction of advanced features from ultrasound images, allowing for the construction of deep models that can automatically analyze and classify pulmonary nodules with a high degree of accuracy.

However, despite these advancements in deep CNNs for pulmonary nodule classification, several limitations persist. One of the primary challenges faced by these models is the diminishing accuracy as network depth increases, often due to issues like the vanishing gradient problem (10). This leads to difficulty in effectively training very deep networks, which in turn hinders their ability to generalize well to new data (11). Furthermore, various architectures, such as AlexNet (12), VGGNet (13), ResNet (14), and DenseNet (15), differ significantly in key aspects like network depth, filter size, and activation functions (16). These architectural variations directly influence each model’s capacity to capture the complex patterns, and intricate relationships present in medical images (17). While shallow networks may struggle with more detailed feature extraction (13), deeper architectures, though capable of capturing finer details, often require careful design to avoid overfitting and performance degradation (11). These limitations highlight the need for a novel deep learning approach tailored to the unique characteristics of pulmonary nodule ultrasound images, enabling the capture of intricate features and addressing challenges like diminishing accuracy, overfitting, and the vanishing gradient problem.

Therefore, to address these limitations, this study proposes a novel deep learning model (DCEF) incorporating deep cross-entropy fusion function. By leveraging the strengths of multiple deep neural networks, our proposed model aims to to overcome the limitations of existing approaches and enhance the classification accuracy of pulmonary nodules in grayscale ultrasound images.

## 2 Materials and methods

### 2.1 Patients

A retrospective collection was conducted on 135 patients who underwent percutaneous pulmonary nodule biopsy under ultrasound guidance in Affiliated People’s Hospital of Jiangsu University from August 2021 to September 2023. Among them, there were 38 cases of benign nodules and 97 cases of malignant nodules, with an age range of 44 to 92 years and an average age of 69.87 ± 9.25 years. Inclusion criteria: 1) Routine ultrasound examination was performed before surgery in our hospital; 2) The nature of the nodules was confirmed by pathological examination after pulmonary needle biopsy; 3) The ultrasound images collected met the criteria for clarity. Exclusion criteria: 1) Incomplete or unclear image data; 2) Unclear pathological results; 3) Presence of other tumors. We collected comprehensive clinical, imaging, and pathological data from patients, including age, gender, lesion diameter, lesion location, pathological type, and presence of distant metastasis (4). The pulmonary nodules in this study ranged from approximately 1 cm to over 7 cm, with malignant nodules averaging 5.04 ± 2.42 cm and benign nodules averaging 3.33 ± 2.19 cm, as detailed in **Table 1**. Given that ultrasound is particularly effective for evaluating medium-to-large nodules, our dataset aligns with previous findings regarding its clinical utility in guided interventions. This study received approval from the Ethics Committee of Affiliated People’s Hospital of Jiangsu University, and all patients provided informed consent.

**Table 1:** Survey of clinical and pathological characteristics.

Variable	Malignant nodules	Benign nodules	P
Age	71.69±8.42	65.21±9.60	<0.001
Gender			0.544
Male	69	25	
Female	28	13	
Lesion diameter (cm)	5.04±2.42	3.33±2.19	0.227
Lesion location			0.039
Right upper lobe	16	13	
Right middle lobe	7	1	
Right lower lobe	21	9	
Left upper lobe	26	12	
Left lower lobe	27	3	
Pathological type			0.799
Peripheral	88	35	
Central	9	3	
Pathological pattern			
Adenocarcinoma	49		
Squamous cell carcinoma	27		
Other	21		
Presence of Distant Metastasis			
Yes	51		
No	46		

### 2.2 Image annotation

**Figure 1:**
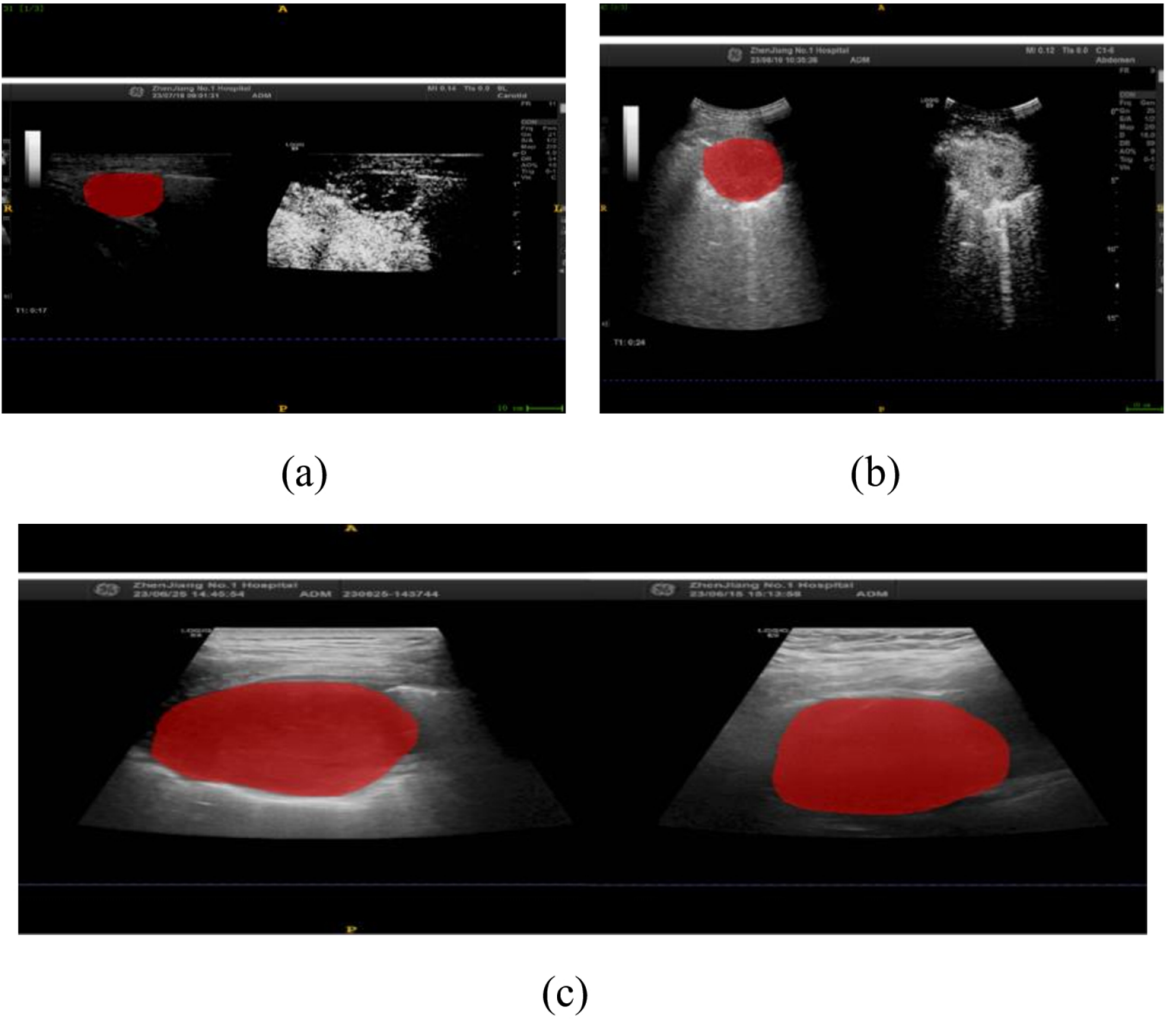
Ultrasound-Guided Puncture of Pulmonary Nodules **(a)** A 67-year-old male patient with left upper peripheral lung adenocarcinoma. **(b)** A 67-year-old male patient with left lower lung central squamous cell carcinoma. The red areas indicate the regions of interest (ROIs) for the punctures. **(c)** Gray-scale ultrasound images of the ROIs for both patients.

In this study, we implemented a meticulously structured annotation process to ensure precise delineation and standardization of Regions of Interest (ROIs) within ultrasound images. All images were initially stored in JPG format and subsequently converted to Nii format using dedicated medical imaging software to maintain consistency across all cases. The ITK-SNAP software was utilized for precise manual segmentation, allowing us to delineate the ROIs along the lesion boundaries, ensuring the focus remained on diagnostically significant regions, as illustrated in **Figure 1**.

A total of 203 ROIs were carefully annotated across 135 patient cases, ensuring that the dataset captured the full spectrum of lesion morphology and variability. These included 57 ROIs from benign nodules and 146 ROIs from malignant nodules, providing a well-balanced representation of the key diagnostic categories. Importantly, the size distribution of the annotated ROIs varied between the two groups, reflecting the natural heterogeneity observed in clinical practice. The average ROI size for benign nodules was 22.4 pixels (range: 15–30 pixels), while malignant nodules had an average ROI size of 23.8 pixels (range: 18–35 pixels). These distinctions align with established pathological characteristics, where malignant nodules often exhibit irregular morphology and slightly larger segmentation areas. To uphold the highest level of accuracy, all ROI annotations were independently reviewed by two senior radiologists with extensive experience in thoracic imaging, and any discrepancies were resolved through expert consensus discussions.

To further contextualize our dataset, the study included 135 patients, comprising 94 males and 41 females, with malignant nodules found to be significantly more prevalent among older individuals (P < 0.001). Other demographic factors, including gender, lesion diameter, and lesion location, did not exhibit statistically significant differences (P > 0.05).

Histopathological evaluation of ultrasound-guided percutaneous pulmonary nodule biopsy confirmed that 97 cases (71.9%) were malignant, encompassing a diverse range of histological subtypes, including adenocarcinoma, squamous cell carcinoma, lung metastasis, small cell carcinoma, sarcomatoid carcinoma, marginal zone B-cell lymphoma, neuroendocrine carcinoma, solitary fibrous tumor, and fibrosarcoma. The remaining 38 cases (28.1%) were benign, diagnosed as chronic pneumonia, granulomatous inflammation, tuberculosis, fibrous tissue hyperplasia, and alveolar epithelial hyperplasia. Additionally, within the 97 malignant cases, 51 patients exhibited distant metastases, while 46 patients did not, further substantiating the clinical heterogeneity of the dataset. Detailed patient characteristics and nodule distributions are systematically provided in **Table 1**.

### 2.3 Experimental parameter settings

The hardware configuration for this experiment was as follows: CPU: Intel® Core™ i7-10700 CPU @ 2.90GHz, RAM: 64G, GPU: NVIDIA GeForce RTX 3080Ti, Operating System: 64-bit Win10. The experiment used the cosine annealing method with adaptive learning rate, with a maximum learning rate of T_max set to 100. During training, the cross-entropy loss function was used for optimization, and the stochastic gradient descent (SGD) optimizer was used with a learning rate of 0.1, momentum parameter of 0.9, weight decay set to 5e-4, batch sample size set to 128, and each training lasted for 100 rounds.

### 2.4 Data preprocessing

To ensure consistency and accuracy in model evaluation, the dataset was divided into training (50%), validation (20%), and test (30%) sets, with 27 benign and 68 malignant nodules in the training and validation sets and 11 benign and 29 malignant nodules in the test set. Several key preprocessing steps were implemented to enhance data uniformity and improve model performance.

Initially, all ultrasound images were converted into a standardized format, ensuring compatibility across different deep learning architectures. Pixel values were normalized to bring them within a suitable range for training, and cropping and resampling were applied to extract the lung region while maintaining a consistent resolution. These steps were essential to prevent biases due to image size variations and to ensure optimal feature extraction by the model.

Given the significant class imbalance between benign and malignant nodules, several techniques were implemented to enhance model robustness. Data augmentation was performed on the minority class (benign nodules) using rotation, flipping, and translation to artificially increase its diversity. Additionally, the Synthetic Minority Over-sampling Technique (SMOTE) was applied to generate synthetic benign samples, helping balance the dataset while preserving meaningful feature distributions. To further refine class distribution, selective undersampling was performed on the malignant class to retain the most diagnostically relevant cases while preventing over-representation.

To compensate for class imbalance during training, class weight adjustments were applied to the loss function, with higher weights assigned to benign nodules (2:1 ratio) to prevent bias toward the majority class. These strategies collectively enhanced the model’s ability to generalize effectively, improving classification performance across both benign and malignant cases.

Following preprocessing, segmentation and annotation were performed using ITK-SNAP software, ensuring precise delineation of lesion boundaries. A total of 203 regions of interest (ROIs) were manually annotated, with 57 ROIs from benign nodules and 146 from malignant nodules. All annotations were independently verified by two expert radiologists, with discrepancies resolved through consensus discussions to maintain annotation accuracy.

These preprocessing steps ensured that the DCEF model was trained on a balanced, high-quality dataset, allowing for robust and reliable classification of pulmonary nodules. By optimizing input representation, class balance, and training stability, this preprocessing pipeline effectively supports the model’s diagnostic performance.

### 2.5 DCEF model architecture

**Figure 2:**
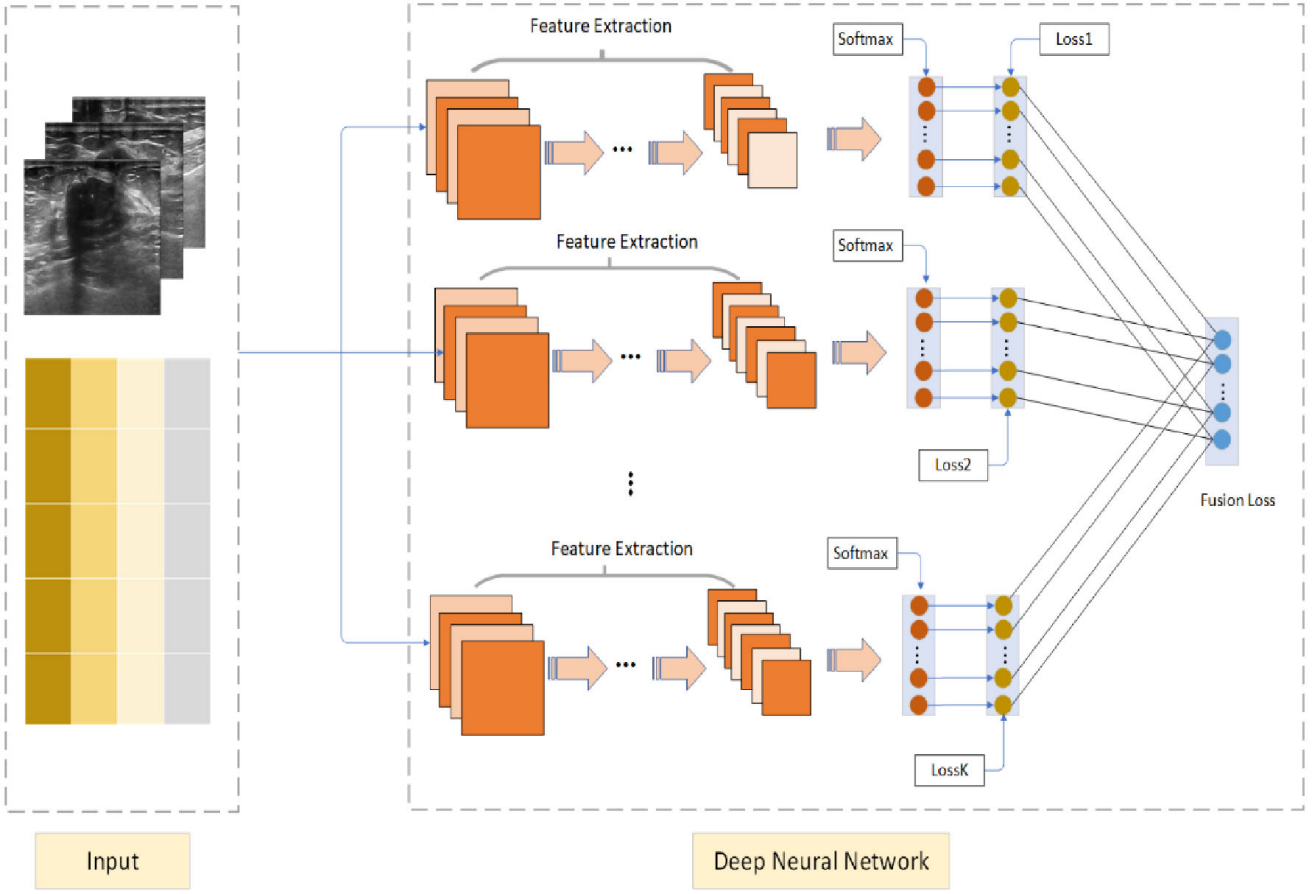
Deep Cross Entropy Fusion (DCEF) model architecture. Multiple CNNs extract features from ultrasound images using convolutional and pooling layers. Extracted features are fed into softmax layers for classification. Individual CNN losses are combined into a unified fusion loss for optimized overall classification accuracy.

The Deep Cross Entropy Fusion (DCEF) model is designed to integrate multiple deep learning architectures, leveraging the strengths of CNN-based feature extraction and Transformer-based attention mechanisms to enhance pulmonary nodule classification. As illustrated in **Figure 2**, the model follows a multi-stream feature extraction approach, where preprocessed ultrasound images are encoded into matrix form and processed independently by different sub-networks. The grids in **Figure 2** represent these encoded ultrasound matrices, allowing the model to systematically analyze spatial and intensity variations in ultrasound images. This structured transformation ensures compatibility across different architectures, optimizing feature extraction and classification performance.

The DCEF model incorporates five sub-networks, each contributing distinct feature extraction capabilities. The CNN-based architectures (ResNet-50, DenseNet-121, VGG-16, and InceptionV3) focus on hierarchical feature learning, capturing spatial patterns and texture variations. Meanwhile, the Vision Transformer (ViT) processes images using self-attention mechanisms, capturing long-range dependencies and global context in ultrasound images. Each sub-network extracts meaningful features independently, before their outputs are integrated through a fusion mechanism. These features are processed through softmax layers, and the model employs a fusion loss function to optimize classification by dynamically weighting each network’s contribution. This ensures that high-performing networks have a stronger influence, improving classification robustness and generalization.

For training and optimization, the model is trained using stochastic gradient descent (SGD) with an initial learning rate of 0.1, momentum of 0.9, and weight decay of 5e-4. A cosine annealing learning rate schedule is applied over 100 epochs, ensuring efficient convergence while preventing overfitting. The combination of CNN-based local feature extraction and Transformer-based global feature modeling allows the DCEF model to effectively differentiate between benign and malignant nodules, leading to superior classification accuracy compared to single-network models.

### 2.6 Experimental methods

The proposed DCEF Model integrates multiple deep learning architectures to enhance feature extraction and classification performance. The cross-entropy loss function is employed to train individual sub-networks and optimize the final fused model by minimizing the discrepancy between predicted and true distributions. Cross-entropy loss is widely used in neural networks for classification tasks, as it effectively guides model training by adjusting network parameters through backpropagation and gradient descent. The standard cross-entropy loss function is defined as:


(1) $$L_{CE}=-\sum_{i=1}^{n}y_{i}\text{log}\frac{e^{x_{i}^{T}w_{j}}}{\sum_{j^{\prime }=1}^{C}e^{x_{i}^{T}w_{j^{\prime }}}}$$

where $x_{i}\in {\mathbb{R}}^{d}$ denotes the deep feature of the *i^th^* sample, $w_{j}\in {\mathbb{R}}^{d}$ denotes the j-th column of the weight $\text{w}\in {\mathbb{R}}^{d\times C}$ , $y_{i}$ represents the true label of the *i^th^* sample, and the class number is C. However, $x_{i}$ Equation 1 represents only a single feature extracted by the network, which is inherently constrained by the network’s capacity and may not capture sufficient feature diversity. Additionally, relying on a single neural network for feature extraction can limit the robustness of learned representations, potentially reducing classification accuracy. To overcome these limitations, our proposed DCEF model integrates multiple sub-networks, including Convolutional Neural Networks (CNNs) and Vision Transformers (ViTs), to extract complementary feature representations from input data. Each sub-network is trained independently to optimize its respective architecture, and their outputs are subsequently fused to generate a more comprehensive and informative prediction. To ensure an effective fusion strategy, we extend the cross-entropy loss function to incorporate multiple sub-networks, defining the DCEF loss function (Equation 2) as:


(2) $$\begin{array}{c} L_{FCE}=-\sum_{k=1}^{K}\pi_{k}\sum_{i=1}^{n}y_{i,k}\log\frac{e^{x_{i,k}^{T}w_{j,k}}}{\sum_{j^{\prime }=1}^{C}e^{x_{i,k}^{T}w_{j^{\prime },k}}}\\ s.t.\;\sum_{k=1}^{K}\pi_{k}=1,\pi_{k}>0 \end{array}$$

where, *x_i,k_* represents the feature extracted by the k-th neural network, and *π_k_* denotes the weight of the k-th network. By using this method, we can integrate the cross-entropy loss functions of multiple sub-networks into a unified fusion loss, thereby optimizing the overall loss. This method adaptively adjusts the weights of the sub-networks, reducing the impact of high-loss sub-networks and enhancing the contribution of low-loss sub-networks, thereby reducing the overall classification loss and improving classification accuracy.

During the *encoding phase*, feature extraction is performed using a combination of CNNs and ViTs, which generate hierarchical feature representations. These extracted features are then processed through a Pyramid Pooling Module (PPM), which enhances multi-scale contextual understanding. The PPM consists of multiple parallel pooling operations with different kernel sizes (e.g., 1×1, 2×2, 3×3, and 6×6), capturing both local and global spatial dependencies. This hierarchical structure enables the model to effectively learn features at multiple scales, making it more robust to variations in tumor size, shape, and texture in ultrasound images. The PPM module serves as a critical component in consolidating spatially enriched feature maps before classification.

In the decoding phase, a Region of Interest (ROI) weight enhancement mechanism is implemented to ensure that the model focuses on diagnostically significant areas of ultrasound images. Given the importance of distinguishing between benign and malignant tumors, it is essential to emphasize the most relevant regions within an image for improved classification. To achieve this, an adaptive weight assignment strategy is introduced using the SoftMax function, which dynamically assigns higher importance to feature responses within the ROI. The weight for each feature response is computed as (Equation 3) follows:


(3) $$W_{i}=\frac{exp\left(F_{i}\right)}{\sum_{j}exp\left(F_{j}\right)}\;$$

where $F_{i}$ represents the feature response at location *i* within the ROI. This computed weight ensures that the most diagnostically relevant regions receive higher attention during classification. The final enhanced feature representation is obtained through a weighted sum operation, where the learned weights $W_{i}$ are used to refine the feature maps (Equation 4):


(4) $$F_{enhanced}=\sum_{i}W_{i}\cdot F_{i}\;$$

By applying this ROI-focused enhancement mechanism, the model effectively prioritizes the most informative regions of an ultrasound image, significantly improving classification accuracy. This ensures that the fused deep learning framework not only leverages multi-network feature diversity but also dynamically refines its attention toward regions that are clinically meaningful.

Therefore, the proposed DCEF model integrates multiple deep networks, optimizes classification using fused cross-entropy loss, and applies region-aware decoding to enhance focus on diagnostically relevant regions, ensuring robust feature extraction and classification.

### 2.7 Optimization of the proposed method

(1) By introducing the Lagrange multipliers η,ξ,τ , we reformulate the constrained optimization problem into an unconstrained framework, as derived in Equations 5–15:


(5) $$\begin{array}{c} argminL\left(\pi ,w_{j,k},\eta ,\xi ,\tau \right)=-\sum_{k=1}^{K}\pi_{k}\sum_{i=1}^{n}y_{i}log\frac{e^{x_{i,k}^{T}w_{j,k}}}{\sum_{j^{\prime }=1}^{c}e^{x_{i,k}^{T}w_{j^{\prime },k}}}+\\ <\eta ,1^{T}\pi -1>+<\xi ,\pi >+\frac{\tau }{2}\left({\left\|1^{T}\pi -1\right\|}_{2}^{2}+{\left\|\pi \right\|}_{2}^{2}\right) \end{array}$$

(2) Update *w_j,k_*


(6) $$J\left(w_{j,k}\right)=-\sum_{k=1}^{K}\pi_{k}\sum_{i=1}^{n}y_{i}log\frac{e^{x_{i,k}^{T}w_{j,k}}}{\sum_{j^{\prime }=1}^{c}e^{x_{i,k}^{T}w_{j^{\prime },k}}}$$

The partial derivative with respect to *w_j,k_* is as follows:


(7) $$\begin{array}{c} J\left(w_{j,k}\right)=-\sum_{k=1}^{K}\pi_{k}\sum_{i=1}^{n}y_{i}log\frac{e^{x_{i,k}^{T}w_{j,k}}}{\sum_{j^{\prime }=1}^{c}e^{x_{i,k}^{T}w_{j^{\prime },k}}}\\ =-\sum_{k=1}^{K}\pi_{k}\sum_{i=1}^{n}y_{i}\left(x_{i,k}^{T}w_{j,k}-log\sum_{j^{\prime }=1}^{c}e^{x_{i,k}^{T}w_{j^{\prime },k}}\right) \end{array}$$

(8) $$\frac{\partial J\left(w_{j,k}\right)}{\partial w_{j,k}}=-\sum_{k=1}^{K}\pi_{k}\sum_{i=1}^{n}y_{i}\left(x_{i,k}-\frac{e^{x_{i,k}^{T}w_{j,k}}\cdot x_{i,k}}{\sum_{j^{\prime }=1}^{c}e^{x_{i,k}^{T}w_{j^{\prime },k}}}\right)$$

(9) $$=-\sum_{k=1}^{K}\pi_{k}\sum_{i=1}^{n}x_{i,k}\left(y_{i}-\frac{e^{x_{i,k}^{T}w_{j,k}}}{\sum_{j^{\prime }=1}^{c}e^{x_{i,k}^{T}w_{j^{\prime },k}}}\right)$$

(3) Update π


(10) $$\begin{array}{c} J\left(\pi \right)=-\sum_{k=1}^{K}\pi_{k}\sum_{i=1}^{n}y_{i}log\frac{e^{x_{i,k}^{T}w_{j,k}}}{\sum_{j^{\prime }=1}^{c}e^{x_{i,k}^{T}w_{j^{\prime },k}}}+\eta 1^{T}\pi +\xi^{T}\pi +\frac{\tau }{2}(\\ -1_{k*1}^{T}\pi -\pi^{T}1_{k*1}+\pi^{T}1_{k*1}1_{k*1}^{T}\pi +\pi^{T}\pi )\; \end{array}$$

The partial derivative with respect to *π* is as follows:


(11) $$\begin{array}{c} \frac{\partial J\left(\pi \right)}{\partial \pi }=-\sum_{i=1}^{n}y_{i}log\frac{e^{x_{i,k}^{T}w_{j,k}}}{\sum_{j^{\prime }=1}^{c}e^{x_{i,k}^{T}w_{j^{\prime },k}}}+\eta 1_{k*1}+\xi +\frac{\tau }{2}(-1_{k*1}\\ -1_{k*1}+2\pi 1_{k*k}+2\pi ) \end{array}$$

Setting $\frac{\partial \text{J}\left(\pi \right)}{\partial \pi }=0$ , we have:


(12) $$\pi =\left(\tau (1_{k*k}+I\right){)}^{-1}\left(\sum_{i=1}^{n}y_{i}log\frac{e^{x_{i,k}^{T}w_{j,k}}}{\sum_{j^{\prime }=1}^{c}e^{x_{i,k}^{T}w_{j^{\prime },k}}}-\eta 1_{k*1}-\xi +\tau 1_{k*1}\right)$$

(4) Update η,ξ,τ :


(13) $$\eta ≔\eta +\theta_{1}\left(1^{T}\pi -1\right)$$

(14) $$\xi ≔\xi +\theta_{2}\pi$$

(15) $$\tau ≔\tau +\frac{\theta_{3}}{2}\left({\left\|1^{T}\pi -1\right\|}_{2}^{2}+{\left\|\pi \right\|}_{2}^{2}\right)$$

### 2.8 Experimental evaluation metrics

To assess the performance of our deep learning model, we employed a suite of five metrics: accuracy, sensitivity, specificity, precision, and F1-score. These metrics provide a comprehensive evaluation of the model’s ability to predict the correct class, identify true positives, exclude true negatives, and maintain a balance between precision and recall.

Accuracy measures the overall correctness of the model’s predictions, while sensitivity and specificity evaluate its ability to detect true positives and exclude true negatives, respectively. Precision assesses the reliability of positive predictions, and the F1-score provides a balanced measure of precision and recall. Below are the formulas for the evaluation metrics, as defined in Equations 6–20:


(16) $$Accuracy=\frac{TP+TN}{TP+TN+FP+FN}$$

(17) $$Sensitivity=\frac{TP}{TP+FN}$$

(18) $$Specificity=\frac{TP}{TN+FP}$$

(19) $$Precision=\frac{TP}{TP+FP}$$

(20) $$F1\_score=\frac{2TP}{2TP+FP+FN}$$

where TP, TN, FP, and FN represent true positives, true negatives, false positives, and false negatives, respectively.

## 3 Results

### 3.1 Clinical data

In this study, a total of 135 patients were included, including 94 male patients and 41 female patients. Compared with the benign nodule group, the malignant nodule group had older patients (P<0.001, statistically significant difference), while gender, lesion diameter, and lesion location showed no statistical significance (P>0.05). The pathological results after ultrasound-guided percutaneous pulmonary nodule biopsy showed 97 cases of malignant nodules (71.9%), including 49 cases of adenocarcinoma, 27 cases of squamous cell carcinoma, 8 cases of lung metastasis, 7 cases of small cell carcinoma, 2 cases of sarcomatoid carcinoma, 1 case of marginal zone B-cell lymphoma, 1 case of neuroendocrine carcinoma, 1 case of solitary fibrous tumor, and 1 case of fibrosarcoma. There were 38 cases of benign nodules (28.1%), including 23 cases of chronic pneumonia, 6 cases of granulomatous inflammation, 3 cases of tuberculosis, and 3 cases of fibrous tissue hyperplasia, and 3 cases of alveolar epithelial hyperplasia. Among the malignant nodules, 51 patients had distant metastasis, while 46 patients did not have distant metastasis. Please refer to **Table 1** for details.

### 3.2 Model performance analysis

**Figure 3:**
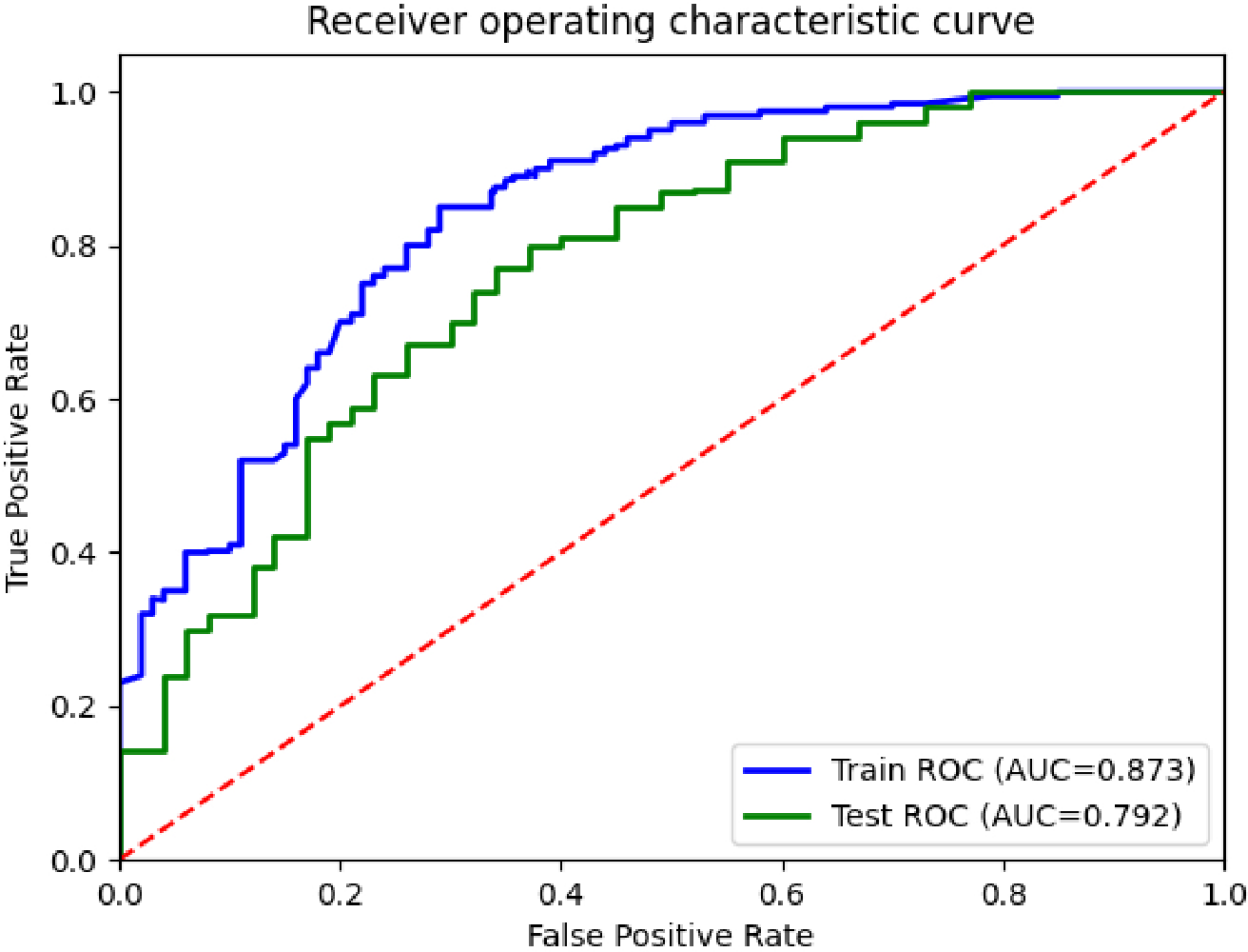
ROC curves for the differentiation of benign and malignant pulmonary nodules in the DCEF classification model.

In this experiment, the performance of the classification model was analyzed and evaluated using receiver operating characteristic (ROC) curves, as shown in **Figure 3**. The figure shows the ROC curves of the training and test sets, corresponding to AUC values of 0.873 and 0.792, respectively. By comparing the two curves in the figure, it can be seen that the performance of the model on the training and test sets was superior to the random classifier (AUC=0.5), confirming the effectiveness and good generalization ability of the model.

### 3.3 Classification performance comparison and analysis

**Table 2** presents the experimental results of various algorithms for pulmonary nodule classification, including XGBoost (18), DeepForest (19), EMA (20), SSE (21), CCE (22), and the proposed DCEF model. The DCEF model consistently outperforms all other algorithms across precision, sensitivity, F1-score, specificity, and accuracy, achieving the highest accuracy of 71.50%. This highlights its effectiveness in distinguishing between benign and malignant pulmonary nodules.

**Table 2:** Experimental results of different algorithms (%).

Methods	Precision	Sensitivity	F1-score	Specificity	Accuracy
XGBoost	0.6491	0.6215	0.6265	0.6541	0.6324
	±0.021	±0.019	±0.033	±0.025	±0.027
DeepForest	0.6177	0.6326	0.6283	0.6023	0.6135
	±0.013	±0.008	±0.025	±0.027	±0.015
EMA	0.6628	0.653	0.6575	0.6892	0.6543
	±0.070	±0.074	±0.062	±0.058	±0.089
SSE	0.7082	0.6928	0.7061	0.6448	0.6882
	±0.051	±0.044	±0.053	±0.061	±0.039
CCE	0.7132	0.6711	0.6933	0.6874	0.6964
	±0.067	±0.084	±0.094	±0.081	±0.073
FCE	0.7255	0.7069	0.7113	0.7058	0.715
	±0.031	±0.042	±0.027	±0.036	±0.062

The table also demonstrates significant improvements achieved by DCEF compared to XGBoost, DeepForest, EMA, SSE, and CCE, indicating its ability to capture relevant features and make accurate predictions. Additionally, the standard deviation values (e.g., ± 0.031 for DCEF’s precision) provide insights into the variability of the results, allowing for a more comprehensive assessment of the model’s performance.

### 3.4 Comparison under different neural networks

Deep learning models have significantly transformed the field of medical image analysis, particularly in pulmonary nodule classification. Convolutional Neural Networks (CNNs) have been a dominant approach due to their ability to learn spatial hierarchies of features, making them effective for lesion detection and classification. More recently, Transformer models originally developed for natural language processing, have gained prominence in computer vision tasks due to their ability to capture long-range dependencies and global contextual information within images. To ensure a balanced and comprehensive evaluation, we selected both well-established CNN architectures and a Transformer-based model as presented in **Table 3**. The CNNs incorporated in our study include VGG-16, ResNet-18, DenseNet-121, and InceptionV3 (23), which have been widely utilized in medical imaging research and serve as established benchmarks for evaluating feature extraction performance. Additionally, we included the Vision Transformer (ViT), a self-attention-based architecture that has demonstrated strong performance in image classification tasks by learning global dependencies more effectively than traditional CNNs.

**Table 3:** Image Prediction Error Rate (%) for Single Networks and Fusion Networks.

VGG16	ResNet18	DenseNet121	InceptionV3	ViT	Error Rate(%)	F1-score(%)
✓					36.25	60.36
	✓				33.37	58.75
		✓			34.58	61.28
			✓		33.54	63.29
				✓	31.36	67.21
✓	✓				32.67	66.01
✓	✓	✓			30.35	68.96
✓	✓	✓	✓		30.13	69.1
✓	✓	✓	✓	✓	**28.81**	**71.13**

Bold values indicate the best (lowest) error rate and highest F1-score.

While the selected architectures provide a strong baseline for evaluating our fusion strategy, we acknowledge the rapid advancements in deep learning and the introduction of newer architectures such as Swin Transformer, ConvNeXt, and EfficientNet. These models have demonstrated impressive performance in general computer vision tasks by integrating transformer-like global dependencies with CNN-based local feature extraction. However, their specific application in medical imaging, particularly pulmonary nodule classification, remains an evolving area of research. Given the need for interpretability and extensive clinical validation, we prioritized architectures with well-documented performance in this domain. Our study focuses on evaluating architectures that have been extensively validated in pulmonary imaging, ensuring that our comparisons are aligned with prior research in the medical community.

**Figure 4:**
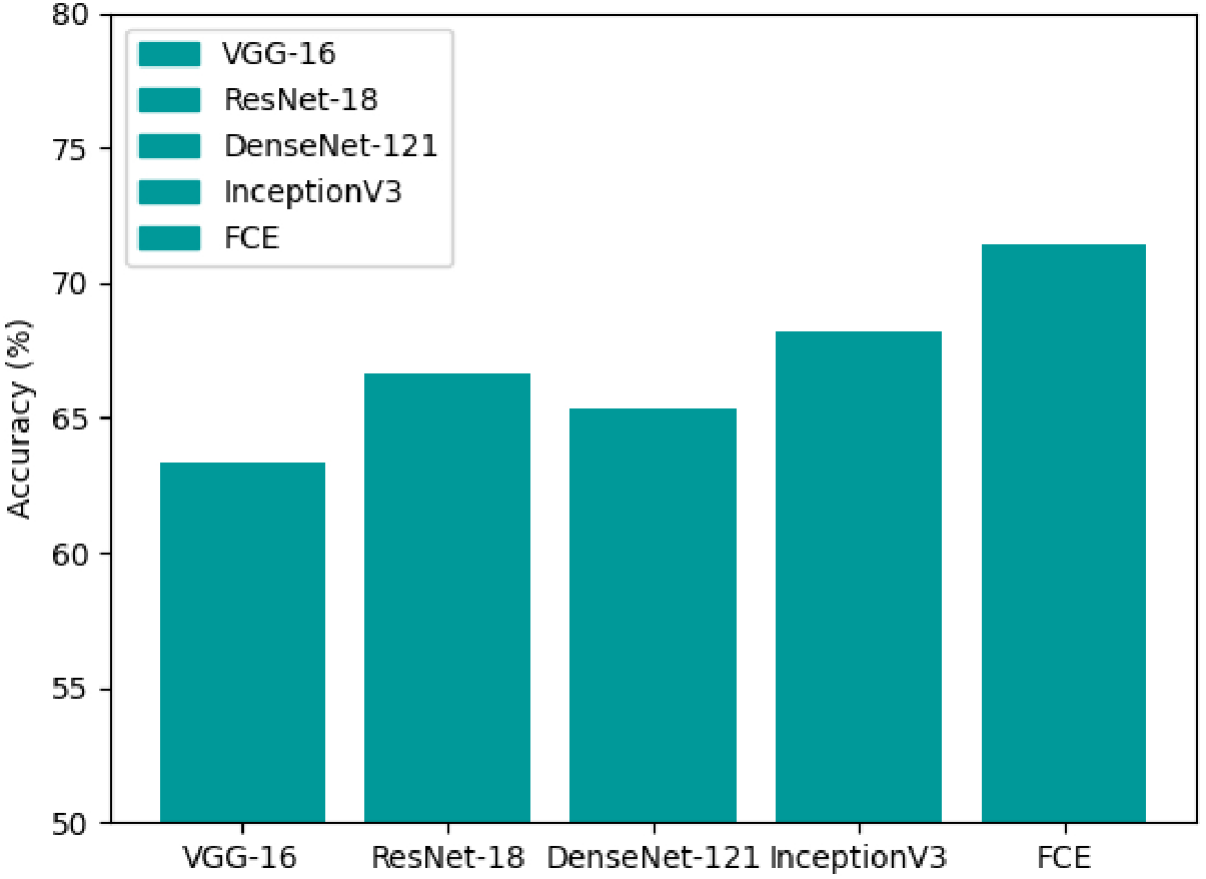
Accuracy comparison of various CNNs and ViT architectures for pulmonary nodule classification.

The classification accuracy of each model, as shown in **Figure 4**, indicates that ViT outperforms traditional CNNs with an accuracy of approximately 68%, reinforcing the effectiveness of self-attention mechanisms in medical imaging tasks. However, our proposed DCEF model achieves the highest accuracy, exceeding 70%, demonstrating the advantages of leveraging multiple architectures in a fusion strategy. In contrast, the other CNN models exhibit accuracy levels ranging from 62% to 68%, with DenseNet-121 and InceptionV3 performing slightly better than VGG-16 and ResNet-18, yet still falling short of the DCEF model’s performance. These results underscore that while established CNN architectures and Transformer models can provide reasonable accuracy, the DCEF model’s ensemble approach significantly enhances classification performance, making it a robust framework for pulmonary nodule analysis.

**Figure 5:**
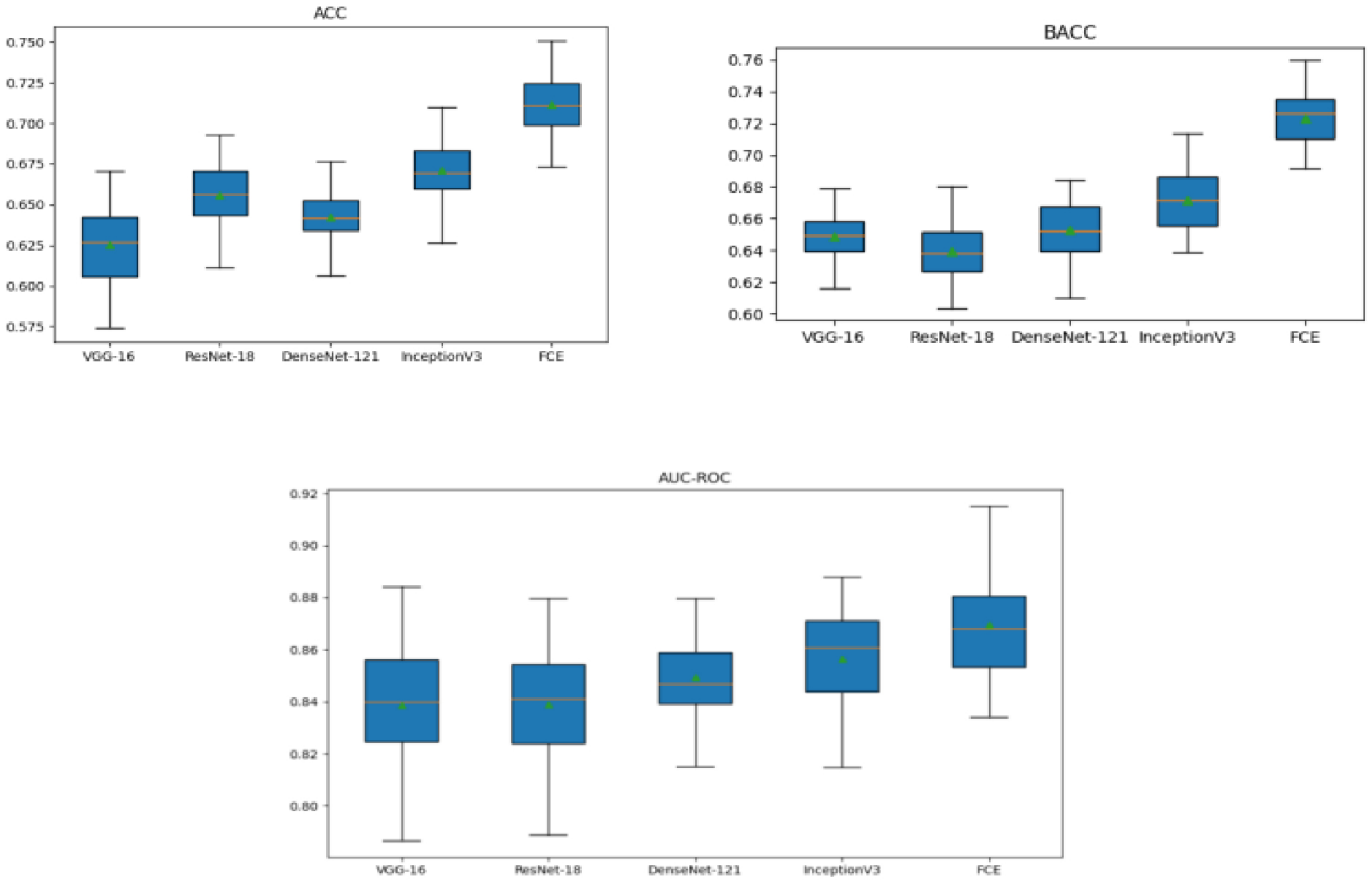
5×5 cross-validation results: ACC, BACC, and AUC-ROC.

To further evaluate model performance, **Figure 5** presents box plots comparing the architectures across three critical evaluation metrics: accuracy (ACC), balanced accuracy (BACC), and area under the receiver operating characteristic curve (AUC-ROC). Each box plot displays the median (green dot), interquartile range (blue box), and variability (whiskers) of each model’s performance. The DCEF model consistently achieves the highest median values for ACC, BACC, and AUC-ROC, reinforcing its superiority in pulmonary nodule classification tasks. Moreover, while ViT demonstrates improved performance over traditional CNNs, the DCEF model maintains higher stability and robustness across multiple runs, confirming the benefits of combining CNN and Transformer architectures in an ensemble learning framework.

We recognize that newer deep learning models such as Swin Transformer, ConvNeXt, and EfficientNet have recently gained prominence in image classification. While these architectures have shown promise in leveraging hybrid CNN-transformer paradigms, their specific utility in pulmonary imaging is still under exploration. As part of our future research, we plan to incorporate these next-generation architectures into our fusion strategy to further assess their potential in medical imaging applications. By integrating advanced hybrid architectures, we aim to enhance the generalizability and robustness of our framework, ensuring its adaptability to evolving deep learning methodologies in medical diagnostics.

### 3.5 Addressing key challenges in model training

**Figure 6:**
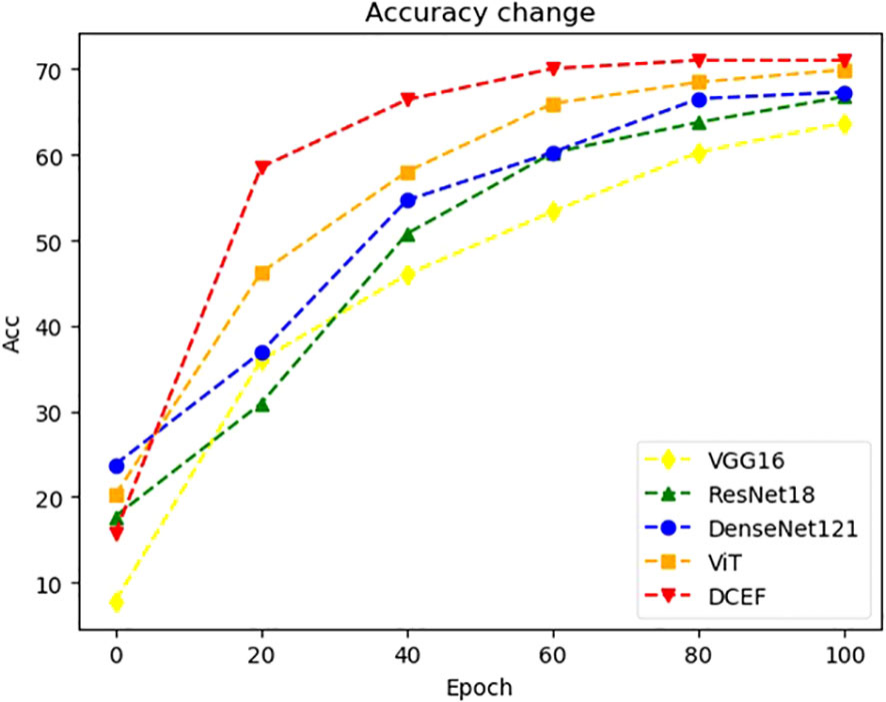
The change trend of training accuracy with the increase of training epoch.

**Figure 7:**
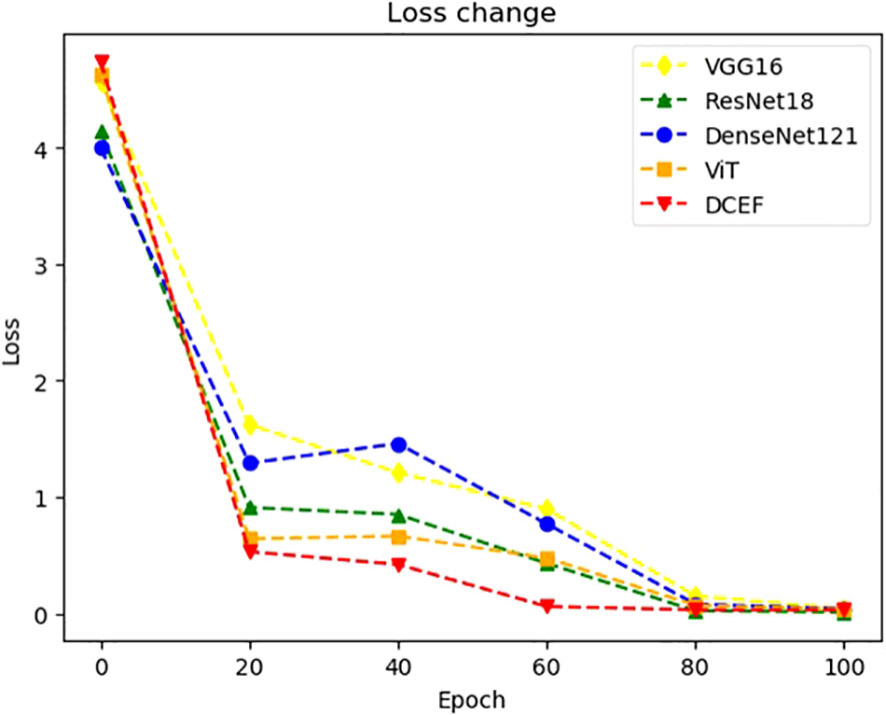
The change trend of training loss with the increase of training epoch.

The DCEF model effectively mitigates diminishing accuracy, overfitting, and the vanishing gradient problem, as demonstrated by the experimental results in **Figures 6**, **7**. Unlike traditional single-model architectures, the DCEF framework integrates multiple CNNs and a Vision Transformer (ViT) model, forming a robust ensemble that enhances feature extraction, representation learning, and classification performance.

#### 3.5.1 Diminishing accuracy with increasing network depth

As shown in **Figure 6**, the accuracy trends indicate that DCEF maintains a steady increase in accuracy throughout training, ultimately surpassing 70 percent accuracy, outperforming individual models. This confirms that the ensemble strategy mitigates the diminishing returns on accuracy, a common issue in deep networks. By combining CNN-based hierarchical feature extraction with the global context awareness of ViT, the DCEF model achieves comprehensive feature learning, reducing the accuracy degradation that typically affects deep networks.

#### 3.5.2 Overfitting mitigation

The loss curves in **Figure 7** provide strong evidence of the DCEF model’s ability to generalize effectively. Unlike individual CNNs or ViT, which exhibit fluctuations indicative of overfitting, the DCEF model demonstrates a stable decline in loss, followed by convergence. The final loss values for DCEF are lower than those of individual networks, indicating an improved trade-off between bias and variance. Furthermore, the close alignment between training and test accuracies suggests that DCEF mitigates overfitting, as it avoids large discrepancies between training and validation performance.

#### 3.5.3 Vanishing gradient problem

The smooth and continuous decline in loss observed in **Figure 7** confirms that DCEF efficiently propagates gradients during backpropagation, preventing stagnation in weight updates. The integration of residual connections from ResNet-based sub-networks and attention mechanisms from ViT ensures that gradients flow effectively, allowing stable convergence even in deeper layers. The absence of abrupt gradient saturation further demonstrates the model’s resilience to the vanishing gradient problem.

### 3.6 Ablation study

To gain a deeper understanding of the specific impact of each network architecture on the experimental results, an ablation study was conducted in this research. The ablation study assesses the contribution of each architecture to the model performance by individually removing or combining different networks. The results of the study are presented in **Table 2**, which shows the image prediction error rates and F1-scores for different network combinations.

The ablation study reveals that the integration of multiple convolutional neural network architectures significantly enhances the model’s performance. As observed from the table, the performance improves with the addition of each network architecture. The standalone InceptionV3 network shows the lowest error rate among individual networks, indicating its strong feature extraction capabilities. However, when combined with other architectures, the ensemble model achieves the best performance, with the lowest error rate of 28.81% and the highest F1-score of 71.13%.

This improvement can be attributed to the complementary strengths of the different architectures. VGG16, known for its simplicity and depth, contributes basic feature extraction capabilities. ResNet18, with its residual connections, helps in alleviating the vanishing gradient problem and enables the training of deeper networks. DenseNet121, through its dense connectivity pattern, enhances feature propagation and reuse. InceptionV3, with its multi-scale feature extraction, captures a wide range of patterns in the data.

The results of the ablation study demonstrate that the proposed DCEF model, by integrating these diverse architectures, effectively captures a comprehensive set of features from ultrasound images. This leads to more accurate and robust classification of pulmonary nodules, highlighting the practical value of the DCEF model in medical imaging applications. The study also underscores the importance of ensemble learning in enhancing the performance of deep learning models for critical tasks such as medical diagnosis.

## 4 Discussions

CNNs have emerged as a transformative tool in medical imaging, particularly for tasks such as disease detection and classification (24). By mimicking the human visual cortex, CNNs extract features from input data using convolutional layers, reduce dimensionality through pooling layers, and generate predictions through fully connected layers (25). Recent advancements in CNN architectures such as ResNet, DenseNet, VGG, and InceptionV3 have significantly improved their performance, allowing for more accurate and efficient disease detection. Previous studies have demonstrated the efficacy of deep learning models in distinguishing between benign and malignant pulmonary nodules using CT images. For example, Heuvelmans et al. (9) developed the Lung Cancer Prediction Convolutional Neural Network (LCP-CNN), achieving an overall AUC of 94.5%, with high accuracy in differentiating between benign and malignant nodules. Similarly, Xie et al. (25) proposed a multi-view knowledge-based collaborative (MV-KBC) deep model for classifying malignant from benign nodules in CT images, achieving an accuracy of 91.60% and an AUC of 0.95. Despite these advancements, there remains a significant gap in research regarding the use of deep learning models for differentiating between benign and malignant pulmonary nodules using lung ultrasound images.

Recent studies, however, have suggested that lung ultrasound images can offer significant advantages for tumor classification. Ultrasound provides real-time, non-invasive imaging, offering excellent soft tissue contrast, which makes it an attractive tool for evaluating pulmonary nodules. For example, Yadav et al. (26) developed an efficient computer-aided diagnostic (CAD) system for thyroid tumor characterization using ultrasound images. Additionally, Du et al. (27) proposed an ultrasound-based multi-class prediction algorithm for ovarian tumor classification. Furthermore, ultrasound imaging can detect subtle changes in nodule characteristics, such as vascularity and echogenicity, which are often challenging to discern using CT scans. These factors suggest that deep learning models trained on lung ultrasound images could potentially achieve comparable or even superior performance in distinguishing benign from malignant pulmonary nodules.

However, ultrasound imaging does have several limitations compared to CT scans and chest X-rays, particularly when considering the use of neural network architectures enhanced with visual attention mechanisms. While ultrasound offers real-time and non-invasive imaging with exceptional soft tissue contrast, it has lower spatial resolution and limited depth penetration compared to CT. These limitations can make it difficult to detect small or deeply located nodules. CT scans, on the other hand, provide high-resolution, cross-sectional images that allow for the detection of subtle lesions, including small nodules. Chest X-rays, though not as detailed as CT, are widely available and offer fast imaging, but they lack the fine resolution needed for detecting early-stage tumors. Another challenge with ultrasound is its high operator dependence. The quality of ultrasound images can vary significantly based on the skill and experience of the technician performing the scan, leading to potential inconsistencies in image quality. In contrast, CT scans and chest X-rays are less sensitive to operator variability, which can contribute to more consistent results across different healthcare settings.

Furthermore, neural network architectures that utilize visual attention mechanisms, which have shown substantial success in CT and chest X-ray imaging, can struggle with ultrasound images. Attention mechanisms help neural networks focus on the most relevant regions of an image, such as tumors or other anomalies, improving classification accuracy. However, ultrasound images are often less uniform in quality, with greater variability in resolution and contrast, which may hinder the effectiveness of these attention-based models. While attention mechanisms work well on high-resolution, consistent images (such as those from CT or X-ray), ultrasound images inherent limitations make it more challenging for these models to accurately identify and focus on key features.

Despite these challenges, ultrasound remains a valuable imaging modality due to its ability to provide real-time results, its non-invasive nature, and its excellent soft tissue contrast. When combined with deep learning models, such as the DCEF model presented in this study, ultrasound can still provide valuable diagnostic information for pulmonary nodule classification.

The DCEF model presented in this study specifically addresses several challenges associated with ultrasound imaging for pulmonary nodule classification. It overcomes the issues of lower spatial resolution, limited depth penetration, and operator dependence that can affect the quality and consistency of ultrasound images. First, the model compensates for lower spatial resolution by leveraging an ensemble strategy that combines multiple CNN architectures, such as ResNet, DenseNet, VGG, and InceptionV3. Each network captures different features from the ultrasound images, enabling the model to aggregate these diverse features and enhance the classification accuracy despite the lower resolution. Second, limited depth penetration is overcome by the model’s ability to fuse information from multiple CNNs, allowing it to capture both superficial and deeper features of the nodules. This multi-network approach improves the model’s ability to identify nodules at varying depths, even when depth penetration in ultrasound images is restricted. Lastly, operator dependence is mitigated by the ensemble approach, as the DCEF model is less sensitive to inconsistencies in image quality that arise from variations in the technician’s skill. The diverse networks in the ensemble focus on different aspects of the ultrasound images, providing a more robust and reliable classification, even when image quality may vary across different scans.

By leveraging an ensemble strategy that combines multiple CNN sub-networks, the DCEF model utilizes cross-entropy loss functions to extract a comprehensive set of features from the input data. One of the main advantages of the DCEF approach is its ability to aggregate predictions from multiple neural networks, resulting in improved accuracy and robustness. The diversity achieved by fusing different CNN architectures allows the model to capture a broader range of patterns within ultrasound images, thereby promoting better generalization and reducing the risk of overfitting. By combining the strengths of multiple CNNs, the DCEF model is able to extract richer and more informative features, offering a more complete representation of the images. This leads to more accurate and confident clarifications, which are critical in medical applications where precision is paramount.

This study evaluated the effectiveness of the DCEF model in differentiating between benign and malignant pulmonary nodules using two-dimensional grayscale ultrasound images. The DCEF model, composed of networks including ResNet, DenseNet, VGG, and InceptionV3, achieved an accuracy of 71.5%, sensitivity of 70.7%, and AUC of 0.87, demonstrating strong diagnostic performance with a high true positive rate and a low false positive rate. These results indicate that the DCEF model is capable of distinguishing between benign and malignant pulmonary nodules with a high degree of reliability. The superior performance of DCEF can be attributed to its ability to effectively combine the strengths of various CNN architectures, each capturing different features of the ultrasound images.

Furthermore, the DCEF model was compared to other state-of-the-art deep learning algorithms, such as XGBoost (18), DeepForest (19), EMA (20), SSE (21), and CCE (22). XGBoost is a highly scalable and efficient machine learning algorithm based on gradient boosting trees. DeepForest utilizes deep learning techniques, including CNNs, for image feature extraction and object classification. EMA achieves continuous feature extraction through an encoder-decoder architecture. SSE trains multiple models in parallel to improve performance, and CCE combines cross-entropy and complementary entropy into a unified training objective, reducing prediction risks for minority classes without requiring additional data augmentation.

From the results, the DCEF model achieved an accuracy of 71.5%, which is 1.86% higher than the CCE algorithm, a sensitivity of 70.69%, 1.41% higher than SSE, a specificity of 70.58%, 1.66% higher than EMA, a precision of 72.55%, 1.23% higher than CCE, and an F1-score of 71.13%, which is 1.8% higher than SSE. These results demonstrate significant improvements across all performance metrics, further confirming the superiority and practicality of the DCEF model in distinguishing between benign and malignant pulmonary nodules.

However, several limitations should be considered in this study. Firstly, the relatively small sample size may affect the generalizability and robustness of the results. To validate the findings and assess the model’s performance across a broader range of patient populations, we will involve larger and more diverse datasets in future research. Secondly, the current study primarily focuses on nodules of varying sizes, but the diagnostic potential of the DCEF model in distinguishing between smaller nodules (≤2 cm) and larger ones (>5 cm) will also be investigated. Nodule size is an important factor in determining malignancy, and exploring how the model performs with different size ranges will enhance its clinical utility. Additionally, we will explore the model’s ability to predict disease metastasis and its application to other types of lung pathology. By addressing these aspects, the model’s clinical relevance and diagnostic accuracy will be further strengthened.

## 5 Conclusions

This study introduced the DCEF model for pulmonary nodule classification using ultrasound imaging. By integrating multiple deep learning architectures, including CNN-based feature extractors (ResNet-50, DenseNet-121, VGG-16, InceptionV3) and a Transformer-based model (ViT), the DCEF model effectively captures both local spatial features and global contextual relationships, enhancing classification performance. The fusion loss mechanism optimally combines features from different sub-networks, ensuring superior accuracy and robustness. Experimental results, including ablation studies, demonstrated that DCEF outperforms conventional CNN-based methods, achieving an AUC of 0.87 and an F1-score of 71.13%, highlighting its effectiveness in pulmonary nodule classification.

Despite these promising results, the study is constrained by the size of the dataset, necessitating further validation on larger, multi-center cohorts. Future research will explore next-generation architectures such as Swin Transformer and ConvNeXt and investigate the integration of multi-modal imaging techniques to enhance diagnostic precision. The proposed DCEF model provides a robust, interpretable, and clinically relevant deep learning framework for ultrasound-based lung cancer detection, offering valuable insights to aid radiologists in early diagnosis and decision-making.

## Data Availability

The original contributions presented in the study are included in the article/supplementary material. Further inquiries can be directed to the corresponding authors.

## References

[1] Li C Lei S Ding L Xu Y Wu X Wang H. Global burden and trends of lung cancer incidence and mortality. Chin Med J (Engl). (2023) 136:1583–90. doi: 10.1097/CM9.0000000000002529 PMC10325747 37027426

[2] Song F Yang Q Gong T Sun K Zhang W Liu M. Comparison of different classification systems for pulmonary nodules: a multicenter retrospective study in China. Cancer Imaging. (2024) 24:15. doi: 10.1186/s40644-023-00634-y 38254185 PMC10801946

[3] Liang G Yu W Liu SQ Xie MG Liu M. The value of radiomics based on dual-energy CT for differentiating benign from Malignant solitary pulmonary nodules. BMC Med Imaging. (2022) 22:95. doi: 10.1186/s12880-022-00824-3 35597900 PMC9123722

[4] Liu J Qi L Wang Y Li F Chen J Cui S. Development of a combined radiomics and CT feature-based model for differentiating Malignant from benign subcentimeter solid pulmonary nodules. Eur Radiol Exp. (2024) 8:8. doi: 10.1186/s41747-023-00400-6 38228868 PMC10792135

[5] Kalra MK Sodickson AD Mayo-Smith WW. CT radiation: key concepts for gentle and wise use. Radiographics. (2015) 35:1706–21. doi: 10.1148/rg.2015150118 26466180

[6] Sabirovna IN Raykhona K. Benefits of ultrasound examination. TADQIQOTLAR. UZ. (2024) 31:95–100.

[7] Brown MV Badiei A Arnold M Jersmann H Sullivan T Fielding D. The diagnostic yield of cone beam computed tomography combined with radial-endobronchial ultrasound for the diagnosis of peripheral pulmonary nodules: systematic review and meta-analysis. Chest Pulmonary. (2024) 2.2:100037. doi: 10.1016/j.chpulm.2024.100037 PMC13417805 42548353

[8] Glielmo P Fusco S Gitto S Zantonelli G Albano D Messina C. Artificial intelligence in interventional radiology: state of the art. Eur Radiol Exp. (2024) 8:62. doi: 10.1186/s41747-024-00452-2 38693468 PMC11063019

[9] Heuvelmans MA van Ooijen PMA Ather S Silva CF Han D Heussel CP. Lung cancer prediction by Deep Learning to identify benign lung nodules. Lung Cancer (Amsterdam Netherlands). (2021) 154:1–4. doi: 10.1016/j.lungcan.2021.01.027 33556604

[10] Glorot X Bengio Y. March. Understanding the difficulty of training deep feedforward neural networks. In: Proceedings of the thirteenth international conference on artificial intelligence and statistics. Sardinia, Italy: JMLR Workshop and Conference Proceedings (2010). p. 249–56.

[11] Goodfellow I Bengio Y Courville A. Deep learning. Cambridge: MIT press (2016). 1:2.

[12] Krizhevsky A Sutskever I Hinton GE. ImageNet classification with deep convolutional neural networks. Commun ACM. (2017) 60:84–90. doi: 10.1145/3065386

[13] Simonyan K Zisserman A. Very deep convolutional networks for large-scale image recognition. arXiv preprint arXiv:1409.1556. (2014). doi: 10.48550/arXiv.1409.1556

[14] He K Zhang X Ren S Sun J. (2016). Deep residual learning for image recognition, in: Proceedings of the IEEE conference on computer vision and pattern recognition (Las Vegas, Nevada: IEEE). pp. 770–8.

[15] Huang G Liu Z van der Maaten L Weinberger KQ. (2017). Densely connected convolutional networks, in: Proceedings of the IEEE conference on computer vision and pattern recognition (Honolulu, HI, USA: IEEE). pp. 4700–8.

[16] Szegedy C Liu W Jia Y Sermanet P Reed S Anguelov D. (2015). Going deeper with convolutions, in: Proceedings of the IEEE conference on computer vision and pattern recognition, (Boston, MA, USA: IEEE). pp. 1–9.

[17] LeCun Y Bengio Y Hinton G. Deep learning. Nature. (2015) 521:436–44. doi: 10.1038/nature14539 26017442

[18] Chen T Guestrin C. (2016). Xgboost: A scalable tree boosting system, in: Proceedings of the 22nd acm sigkdd international conference on knowledge discovery and data mining, (San Francisco, CA, USA: Association for Computing Machinery (ACM)). pp. 785–94.

[19] Zhou ZH Feng J. Deep forest. Natl Sci Rev. (2019) 6:74–86. doi: 10.1093/nsr/nwy108 34691833 PMC8291612

[20] Shanahan M Kaplanis C Mitrović J. Encoders and ensembles for task-free continual learning. arXiv preprint arXiv:2105.13327. (2021). doi: 10.48550/arXiv.2105.13327

[21] Huang G Li Y Pleiss G Liu Z Hopcroft JE Weinberger KQ. Snapshot ensembles: Train 1, get m for free. arXiv preprint arXiv:1704.00109. (2017). doi: 10.48550/arXiv.1704.00109

[22] Kim Y Lee Y Jeon M. Imbalanced image classification with complement cross entropy. Pattern Recognition Lett. (2021) 151:33–40. doi: 10.1016/j.patrec.2021.07.017

[23] Wang C Chen D Hao L Liu X Zeng Y Chen J. Pulmonary image classification based on inception-v3 transfer learning model. IEEE Access. (2019) 7:146533–41. doi: 10.1109/Access.6287639

[24] Vainio T Mäkelä T Savolainen S Kangasniemi M. Performance of a 3D convolutional neural network in the detection of hypoperfusion at CT pulmonary angiography in patients with chronic pulmonary embolism: a feasibility study. Eur Radiol Exp. (2021) 5:1–12. doi: 10.1186/s41747-021-00235-z 34557979 PMC8460693

[25] Xie Y Xia Y Zhang J Song Y Feng D Fulham M. Knowledge-based collaborative deep learning for benign-malignant lung nodule classification on chest CT. IEEE Trans Med Imaging. (2019) 38:991–1004. doi: 10.1109/TMI.2018.2876510 30334786

[26] Yadav N Dass R Virmani J. Deep learning-based CAD system design for thyroid tumor characterization using ultrasound images. Multimedia Tools Appl. (2024) 83:43071–113. doi: 10.1007/s11042-023-17137-4

[27] Du Y Guo W Xiao Y Chen H Yao J Wu J. Ultrasound-based deep learning radiomics model for differentiating benign, borderline, and Malignant ovarian tumours: a multi-class classification exploratory study. BMC Med Imaging. (2024) 24:89. doi: 10.1186/s12880-024-01251-2 38622546 PMC11020982

